# Disease Latency according to Asbestos Exposure Characteristics among Malignant Mesothelioma and Asbestos-Related Lung Cancer Cases in South Korea

**DOI:** 10.3390/ijerph192315934

**Published:** 2022-11-29

**Authors:** Da-An Huh, Woo-Ri Chae, Yun-Hee Choi, Min-Sung Kang, Yong-Jin Lee, Kyong-Whan Moon

**Affiliations:** 1 Institute of Health Sciences, Korea University, Anam-ro 145, Seongbuk-gu, Seoul 02841, Republic of Korea; 2 Department of Health and Safety Convergence Science, Korea University, Anam-ro 145, Seongbuk-gu, Seoul 02841, Republic of Korea; 3 BK21 FOUR R&E Center for Learning Health System, Korea University, Anam-ro 145, Seongbuk-gu, Seoul 02841, Republic of Korea; 4 Asbestos Environmental Health Center, Soonchunhyang University Cheonan Hospital, Soonchunhyang 6-gil 31, Dongnam-gu, Cheonan-si 31151, Republic of Korea; 5 Department of Occupational & Environmental Medicine, Soonchunhyang University, Soonchunhyang 6-gil 31, Dongnam-gu, Cheonan-si 31151, Republic of Korea; 6 Department of Health and Environmental Science, Korea University, Anam-ro 145, Seongbuk-gu, Seoul 02841, Republic of Korea

**Keywords:** asbestos, latency period, lung cancer, malignant mesothelioma

## Abstract

Korea was one of the major consumers of asbestos in the late 1900s, and asbestos-related disease patients have been reported continuously to date, owing to long disease latency. Several studies have been conducted to predict the future incidence of malignant mesothelioma and lung cancer in Korea, but little is understood about the latency time. Therefore, the aim of this study is to estimate the latency period of malignant mesothelioma and asbestos-related lung cancer in Korea and its determinants. We obtained information from the Environmental Health Centers for Asbestos in Korea on the history of asbestos exposure and demographic characteristics of 1933 patients with malignant mesothelioma and asbestos-related lung cancer. In our study, the latency periods for malignant mesothelioma and lung cancer were 33.7 and 40.1 years, respectively. Regardless of the disease type, those with a history of exposure related to the production of asbestos-containing products or asbestos factories had the shortest latency period. In addition, we observed that those who worked in or lived near asbestos mines tended to have a relatively long disease latency. Smoking was associated with shorter latency, but no linear relationship between the lifetime smoking amount (expressed in pack years) and latent time was observed. In addition, the age of initial exposure showed a negative linear association with the latency period for mesothelioma and lung cancer.

## 1. Introduction

Asbestos has been widespread worldwide since industrialization [1] and was considered an essential mineral for many activities until health problems caused by asbestos were reported [2]. The effect of asbestos exposure has been demonstrated by many previous studies [3,4,5,6,7,8]; it is now well-known that asbestos exposure is a risk factor for asbestos-related cancers, particularly malignant mesothelioma and lung cancer [9]. An estimated 30,000 mesothelioma deaths were reported by the Global Burden of Diseases 2016 [10], with approximately 180,000 estimated lung cancer deaths attributed to asbestos [11].

Korea was one of the major consumers of asbestos in the late 1900s; approximately 2 to 2.4 million tons of asbestos were mined or imported. Most was used as building materials, such as slate and thermal insulation materials, which cause public health problems associated with asbestos in Korea [12,13,14]. The Ministry of Environment of Korea enacted the Asbestos Injury Relief Act in 2011 and designated two Environmental Health Centers for Asbestos to operate a health surveillance system to identify asbestos victims [15].

Some previous studies conducted in Korea attempted to predict the number of patients with malignant mesothelioma and asbestos-related lung cancer in the future using statistical models such as the Poisson regression or age–period–cohort (APC) model [16,17,18]. To improve the reliability of estimation, it is essential to properly set the latency period for asbestos-related diseases, as well as the accuracy of the model. However, the latency periods for malignant mesothelioma and asbestos-related lung cancer are highly variable [19,20]. In addition, few studies have investigated the putative role of factors such as asbestos exposure pattern and demographic characteristics, which could determine the period between asbestos exposure and the onset of malignant mesothelioma and lung cancer [21,22]. Therefore, a deeper understanding of the factors could help improve predictions of future case of disease.

The aim of this study is to estimate the latency period and its determinants of malignant mesothelioma and asbestos-related lung cancer using information collected by the Ministry of Environment and the Environmental Health Centers for Asbestos. We analyzed the correlation between latency period and asbestos exposure patterns and investigated the presumptive influence of demographic characteristics on latency.

## 2. Materials and Methods

### 2.1. Data Source and Study Population

In this study, we used information about malignant mesothelioma and asbestos-related lung cancer patients collected by the Ministry of Environment and the Soonchunhyang University Cheonan Hospital, one of the Environmental Health Centers for Asbestos in Korea. Under the Asbestos Damage Relief Act, the Ministry of Environment collects information on victims who have experienced occupational or environmental exposure to asbestos through the following two processes: (1) Individuals who were claiming compensation for disease due to asbestos exposure provided information such as asbestos exposure and medical history to the local government; (2) Korean adults are required to undergo regular health examinations under the National Health Insurance Act, and medical institutions have to report to the Ministry of Environment if symptoms suspected as asbestos damage are found in examination subjects. In addition, the Center has been investigating since 2009 to find victims of environmental exposure. The Center classifies areas within 2 km of the asbestos exposure sources (asbestos mine, asbestos industries, shipyard, asbestos containing building, and others) as presumed exposure areas and has conducted health surveys and epidemiologic study of people who have lived in those areas for more than 10 years [15]. This process was carried out in two stages: a primary screening and a detailed examination. The primary screening included a physical examination by an occupational and environmental physician, chest radiography, and interviews using a structured questionnaire. Close examinations, including a computed tomographic scan and a pulmonary function test, were performed on subjects with abnormal findings in the primary screening.

**Figure 1:**
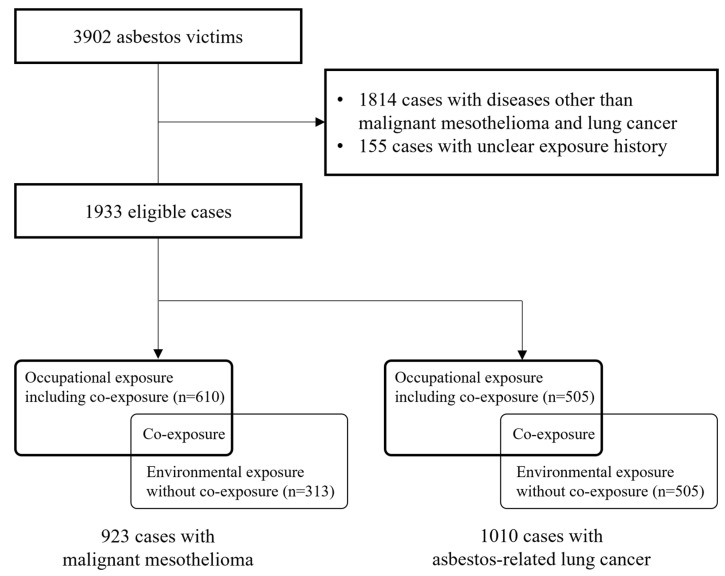
Flow chart describing the selection and exclusion process of study participants.

The Korea Environmental Industry and Technology Institute, an affiliate of the Ministry of Environment, analyzed the causal relationship between asbestos exposure and disease development through the given information and the medical findings. We accessed data on 3902 people who were approved as asbestos victims by 2021 according to this procedure. After excluding patients with unclear exposure history or with diseases other than malignant mesothelioma and lung cancer, 1933 cases were eligible for analysis (Figure 1).

The institutional review board of Soonchunhyang University Cheonan Hospital approved the collection and utilization of data for this study (2009-04-001).

### 2.2. History of Asbestos Exposure

Information on lifetime asbestos exposure was obtained by well-trained researchers of the Environmental Health Center using structured questionnaires developed by the Ministry of Environment. Occupational asbestos exposure was defined as occupational contact with asbestos fibers for at least one year. In addition, data on the name of the workplace, job type, working duration, and first exposure age were collected. To minimize the information bias that may occur during the survey process, the participants’ responses were compared with past records about the location and operating period of the workplace. The types of occupations were classified into three categories: extraction work (asbestos extraction, conveyance, and grinding), production of asbestos-containing products (cements, slates, and fabrics), and maintenance work (demolition and repair of asbestos-containing buildings or equipment).

Environmental exposure to asbestos was defined as non-occupational contact with airborne asbestos fibers caused by exposure sources such as asbestos mines, industries, and loading spaces. The Environmental Health Center collected data about the region of residence, type of exposure sources, distance from the sources, residential duration, first exposure age, and soil cultivation experience. To verify the accuracy of the exposure information provided by the participants, the survey responses were compared with data on Korea’s past exposure sources of asbestos collected by the Ministry of Environment and the participants’ residential registration documents.

We also classified patients who experienced both occupational and environmental exposure to asbestos into the co-exposure group. However, because occupational asbestos exposure levels are generally higher than environmental exposure levels, co-exposure was considered an occupational exposure group in the analysis in this study (Figure 1).

### 2.3. Latency Period

We defined the latency periods as the time between initial exposure to asbestos and disease diagnosis calculated based on the survey results. For occupationally exposed participants, the age at which they started working was considered the initial exposure time. For environmental exposed cases, the year that they began to live near the exposure sources or the exposure sources began to operate was considered the first exposure year.

### 2.4. Statistical Analysis

We performed a univariate analysis to calculate the latency period by age and gender of participants. The mean and median are presented as point estimates, and standard deviation, range, and 5th and 95th percentiles were calculated to evaluate the variability of the latency period. Differences in mean latency period according to gender and age group were assessed using a *t*-test or analysis of variance (ANOVA).

The adjusted latency periods for malignant mesothelioma and lung cancer were calculated using an analysis of covariance (ANCOVA). Gender (male, female), age (continuous), smoking status (never, past smoker, or current smoker), asbestos exposure modalities (occupational, environmental), and age of first exposure (continuous) were considered covariates.

We also investigated the association between the latency period and each factor using multiple linear regression analysis. In the occupational exposure model, the regression coefficient of the exposure period, the initial exposure age, and the pack years were calculated. In addition, the variable of distance from the exposure source to the residence was further considered in the environmental exposure model. In each model, gender and age were considered covariates.

## 3. Results

The general characteristics of participants are presented in Table 1. The number of men was higher than that of women in both diseases. In the case of malignant mesothelioma, more participants were exposed to asbestos occupationally than environmentally, but there was no difference in lung cancer. The number of diagnosed people has increased over time, regardless of the type of disease.

Each latency period was a normal distribution, and the mean (standard deviation) was 33.7 (13.8) years in malignant mesothelioma and 40.1 (16.3) years in lung cancer (Table 2). The latency periods did not significantly differ with respect to gender but tended to increase according to age; the same results were obtained after adjusting for covariates (Table 3).

The adjusted mean latency periods are shown in Table 3. In malignant mesothelioma, the latency was shorter among current smokers and participants occupationally exposed to asbestos. In particular, those who produced asbestos-containing products and those who lived near asbestos factories had the shortest latency. On the other hand, the latency period of those who mined asbestos or lived near asbestos mines was longer than that of other groups. In lung cancer, the latency period is not significantly related to smoking. However, similar to malignant mesothelioma, people occupationally exposed to asbestos had a shorter latency period, whereas those with exposure history related to asbestos mines had longer latency periods than other groups.

All models were adjusted for sex, age, smoking status, asbestos exposure modalities, and age of first exposure.

Table 4 shows the results of multiple regression analysis to evaluate the correlation between latency and variables. After controlling for covariates, the distance from the exposure source to the residence, the exposure duration, and the smoking pack years did not have a linear association with the latency period. However, as expected, the first asbestos exposure age was associated with latency. A one-year increase in first exposure age (range: 1–75) reduced the latency period by about one year, regardless of the type of disease.

Models of occupational exposure were adjusted for age, exposure duration, age of first exposure, and pack years. Models of environmental exposure were adjusted for age, distance from the exposure source to the residence, exposure duration, age of first exposure, and pack years.

## 4. Discussion

The aim of this study was to estimate the latency period of malignant mesothelioma and asbestos-related lung cancer cases in South Korea and its determinants. A total of 1933 cases collected by the Ministry of Environment and the Environmental Health Centers for Asbestos were used in the analyses. In our study, the latency periods for malignant mesothelioma and lung cancer were 33.7 and 40.1 years, respectively. The latency of patients exposed to occupational asbestos was shorter in mesothelioma and lung cancer than in patients exposed to environmental asbestos. Regardless of the type of disease, those who produced asbestos-containing products had the shortest latency period, and those who worked in asbestos mining had longer latency periods than other occupational groups. In cases with environmental asbestos exposure, people who lived near asbestos industries tended to have a shorter latency period. In contrast, those who lived near asbestos mines had a longer latency period. In addition, the age of initial exposure showed a negative linear association with the latency period for mesothelioma and lung cancer.

Asbestos consumption in Korea peaked in 1992 [23], and patients with asbestos-related diseases have been reported continuously until recently, owing to the long latency period. Although all types of asbestos were banned in Korea in 2009 and the risk of asbestos exposure and the epidemiologic characteristics of asbestos-related diseases have been studied in Korea [24,25,26,27,28,29], little is understood about the latency period of malignant mesothelioma and lung cancer in Korea. Some preliminary studies predicted the number of future victims of asbestos-related disease in Korea for asbestos-induced injury compensation [16,17,18]. For example, Kim et al., considered the latency period of asbestos-related diseases as 33 years based on prior global studies that predicted that the number of victims would peak in the early 2020s [16]. However, in our study, the latency period of lung cancer was about 6 years longer than that of malignant mesothelioma, and the period of occupational asbestos exposure was shorter than that of environmental exposure, indicating that latency may vary depending on the type of disease and asbestos exposure pattern.

In this study, participants occupationally exposed to asbestos had a shorter latency period, which is consistent with results reported in previous studies [21,30], indicating that more heavy asbestos exposure may shorten the latency period. However, previously reported results on the association between disease latency and duration or degree of asbestos exposure are inconsistent. In a study on British naval shipyard workers conducted by Hilliard et al., workers who were continuously exposed to asbestos had shorter latency times than workers exposed intermittently [31]. On the other hand, Frost found no evidence that higher-intensity asbestos exposure could shorten the latency period [22]. No association was observed between the exposure duration and the disease latency in our study, possibly because the asbestos fiber levels to which the patients were exposed were not considered in the analysis. It is not easy to obtain data on the fiber levels in asbestos industries because few measurements of asbestos concentration have been carried out in Korea. Therefore, future studies should limit the analysis to a small number of industries with relatively sufficient data and use the average fiber levels in each industry.

We compared the disease latency of each group by further subdividing the type of occupation and exposure source in our previous study [27]. Those who worked to produce asbestos-containing products and those who lived near asbestos industries had a shorter latency than other groups. On the other hand, the latency time was longer for those who experienced asbestos mining-related occupational and environmental asbestos exposure. The toxicity of asbestos differs depending on the type, and it is generally known that chrysotile is less harmful than crocidolite and amosite [19]. According to the records of asbestos use in Korea, the use of crocidolite and amosite was high in asbestos factories [32]. Kim reported that about 40% of asbestos factory workers were exposed to crocidolite [33]. On the other hand, because most of the asbestos produced in Korea is chrysotile [34], it can be expected that the asbestos exposure patterns of miners were different from those of asbestos factory workers. Therefore, it is likely that exposure to crocidolite and amosite, which is more hazardous, could result in a reduced disease latency. We also noted the effect of smoking on latency time. In the case of malignant mesothelioma, it appears that the latency period of current smokers is 2–3 years shorter than that of non-smokers and former smokers, but a linear relationship between latency and pack years was not observed. The latency period for people with fewer than 10 pack years was 35.8 years, which decreased by about 2 years in participants with 10 or more pack years (Table 3). However, with increased pack years, no decreasing latency trend was observed. These results may imply a non-linear relationship between the smoking amount and disease latency caused by asbestos exposure. However, the association between smoking and decreased latency has not been confirmed in many previous studies [22,35], and the smoking status data used in this study cover a limited sample size (169 and 298 cases for malignant mesothelioma and lung cancer, respectively). Therefore, further studies are needed on the effect of smoking on the disease latency from asbestos exposure.

Epidemiological studies report that it takes at least 10 years for most people to develop malignant mesothelioma and lung cancer after initial asbestos exposure [19,36]. However, in some studies, cases with a latency of fewer than 10 years for malignant mesothelioma are often observed [21,22,37]. In our study, four cases of malignant mesothelioma and two cases of lung cancer had a latency period of fewer than 10 years. These cases may be associated with inaccurate information about their history of asbestos exposure, i.e., early exposure to asbestos that they were unaware of. However, because the researchers compared the asbestos exposure histories provided by the participants with historical records, we can consider another possibility, which is that a genetic mutation in the BAP1 tumor suppressor gene predisposes individuals to malignant mesothelioma and lung cancer [38]. Although the importance of mutations as a factor influencing susceptibility to asbestos-related diseases is gradually increasing [39,40,41], studies on BAP1 mutations have not yet been conducted in Korea. Therefore, national examinations are needed to identify potential links between asbestos-related diseases and mutations.

Finally, regardless of the latency period, it is worth noting the proportion of lung cancer patients due to environmental asbestos exposure in our study. According to Table 1, 50% of lung cancer patients experienced environmental asbestos exposure, which is higher than that reported in previous studies. We previously performed a case–control study to evaluate the risk of asbestos exposure to lung cancer in Korea [27]. The odds ratio of environmental asbestos exposure to lung cancer was 1.03 (95% confidence interval: 0.38, 2.77), which means that the increased risk of lung cancer due to environmental exposure is not statistically significant. However, when the study population was separated based on distance from asbestos exposure sources, the lung cancer risk of participants who lived ≤1 km and ≤0.5 km from the asbestos exposure sources were observed to increase by 3.53 times and 6.21 times, respectively. Furthermore, the risk of lung cancer was observed to be 4.47 times higher in those who experienced cultivation near asbestos exposure sources. The Ministry of Environment reported that the soil near asbestos exposure sources was contaminated with asbestos, and Korea had a relatively high proportion of farmers in the 1990s. We believe that these reasons could explain the high proportion of lung cancer patients reported in our study due to environmental asbestos exposure.

## 5. Conclusions

In this study, we analyzed the latency period of malignant mesothelioma and asbestos-associated lung cancer cases in Korea and its determinants. We found that occupational asbestos exposure has a shorter disease latency than environmental exposure. Furthermore, the period can be significantly shortened depending on factors such as asbestos exposure patterns, type of job, type of asbestos exposure source, and initial exposure age. This finding indicates the need for further investigation of asbestos exposure patterns and the development of a response strategy to address asbestos-related diseases. Given the increasing number of diseases caused by exposure to asbestos in Korea, further investigations and prospective studies on disease latency are warranted.

## Figures and Tables

**Table 1:** Characteristics of study population with malignant mesothelioma and lung cancer.

Variable	Malignant Mesothelioma	Lung Cancer
	*n* (%)	*n* (%)
Total	923 (100.0)	1010 (100.0)
Sex		
Male	616 (66.7)	721 (71.4)
Female	307 (33.3)	289 (28.6)
Age		
<60	112 (12.1)	67 (6.6)
60–69	206 (22.3)	244 (24.2)
70–79	289 (31.3)	363 (35.9)
80–89	235 (25.5)	270 (26.7)
≥90	81 (8.8)	66 (6.5)
Smoking status		
Never	80 (8.7)	149 (14.8)
Past smoker	68 (7.4)	109 (10.8)
Current smoker	21 (2.3)	40 (4.0)
Unknown	754 (81.7)	712 (70.5)
Exposure modalities		
Occupational	610 (66.1)	505 (50.0)
Environmental	313 (33.9)	505 (50.0)
Diagnosis year		
<2000	10 (1.1)	1 (0.1)
2000–2004	83 (9.0)	14 (1.4)
2005–2009	174 (18.9)	55 (5.4)
2010–2014	254 (27.5)	213 (21.1)
2015–2019	293 (31.7)	530 (52.5)
≥2020	109 (11.8)	197 (19.5)

**Table 2:** Descriptive statistics on latency period of malignant mesothelioma and lung cancer.

Variable	*n*	Mean Latency (±SD)	*p*-Value	Median Latency	Range (min–max)	5–95 Percentile
**Malignant mesothelioma**						
Total	923	33.7 (±13.8)		34.0	8.0–84.0	14.0–57.8
Sex						
Male	616	33.6 (±13.3)	0.619	33.0	8.0–77.0	14.0–56.0
Female	307	34.1 (±14.5)		34.0	8.0–84.0	15.0–61.0
Age						
<60	112	25.3 ^a^ (±10.9)	<0.001	25.0	8.0–51.0	11.7–44.4
60–69	206	29.9 ^b^ (±11.4)		30.0	10.0–62.0	13.0–49.0
70–79	289	35.2 ^c^ (±13.0)		36.0	8.0–72.0	15.0–57.5
80–89	235	38.6 ^d^ (±14.2)		38.0	11.0–84.0	16.0–68.0
≥90	81	36.3 ^cd^ (±15.5)		37.0	10.0–75.0	13.1–65.6
**Lung cancer**						
Total	1010	40.1 (±16.3)		39.0	7.0–94.0	15.0–73.5
Sex						
Male	721	40.7 (±16.1)	0.076	40.0	7.0–87.0	15.0–73.0
Female	289	38.7 (±16.7)		37.0	11.0–94.0	15.5–74.0
Age						
<60	67	29.4 ^a^ (±10.7)	<0.001	28.0	11.0–54.0	14.0–45.2
60–69	244	32.6 ^a^ (±13.3)		34.0	10.0–65.0	14.0–56.0
70–79	363	41.2 ^b^ (±14.8)		40.0	7.0–75.0	18.0–69.0
80–89	270	45.8 ^c^ (±17.1)		43.0	10.0–84.0	20.0–78.0
≥90	66	49.3 ^c^ (±19.4)		46.5	13.0–94.0	18.0–86.0

Same letter indicates statistical insignificance based on Duncan’s multiple comparisons.

**Table 3:** Adjusted mean latency periods according to characteristics of the participants.

Variable	Malignant Mesothelioma			Lung Cancer		
	N	Estimate (95% CI)	*p*-Value	N	Estimate (95% CI)	*p*-Value
Sex						
Male	616	34.2 (33.1, 35.3)	0.100	721	40.0 (39.6, 40.4)	0.394
Female	307	35.0 (34.0, 36.0)		289	40.2 (39.7, 40.8)	
Age						
<60	112	15.3 (13.8, 16.9)	<0.001	67	23.6 (22.4, 24.8)	<0.001
60–69	206	27.6 (26.5, 28.8)		244	32.5 (31.9, 33.2)	
70–79	289	36.6 (35.5, 37.6)		363	40.6 (40.0, 41.2)	
80–89	235	43.9 (42.6, 45.1)		270	48.4 (47.8, 49.1)	
≥90	81	49.5 (47.8, 51.2)		66	54.8 (53.6, 56.0)	
Smoking status						
Never	80	35.4 (33.9, 36.8)	0.011	149	40.3 (39.7, 40.9)	0.875
Past smoker	68	36.4 (33.6, 39.1)		109	39.9 (39.2, 40.7)	
Current smoker	21	33.3 (31.7, 34.8)		40	40.0 (38.9, 41.2)	
Unknown	754	33.4 (32.9, 33.9)		712	40.2 (39.9, 40.5)	
Lifetime smoking (in pack years)						
<10	93	35.8 (34.4, 37.1)	0.019	174	40.3 (39.7, 40.9)	0.502
10–30	27	33.5 (31.1, 35.9)		52	39.5 (38.4, 40.5)	
30–50	32	33.0 (30.8, 35.2)		52	39.9 (38.9, 41.0)	
≥50	17	33.6 (30.6, 36.7)		20	41.1 (39.5, 42.8)	
Unknown	754	33.4 (32.9, 33.9)		712	40.2 (39.9, 40.5)	
Exposure modalities						
Occupational	610	33.4 (32.4, 34.4)	<0.001	505	39.5 (39.0, 40.0)	<0.001
Environmental	313	35.8 (34.7, 36.9)		505	40.7 (40.2, 41.2)	
Type of job						
Building ^1^	313	32.7 (31.6, 33.7)	<0.001	190	37.7 (36.9, 38.5)	0.009
Production ^2^	54	31.3 (29.7, 33.0)		42	36.7 (35.4, 38.0)	
Maintenance ^3^	143	33.3 (32.1, 34.6)		135	37.9 (37.1, 38.8)	
Mining ^4^	17	34.9 (32.3, 37.6)		72	38.4 (37.4, 39.5)	
Others	83	33.6 (32.2, 35.0)		66	37.3 (36.3, 38.3)	
Type of exposure source						
Asbestos mines	37	40.4 (38.2, 42.5)	<0.001	151	43.5 (42.6, 44.3)	0.238
Asbestos industries	162	33.0 (31.7, 34.3)		255	41.3 (40.6, 42.1)	
Shipyards	25	37.3 (35.1, 39.5)		71	42.5 (41.4, 43.6)	
Asbestos-containing building	62	36.2 (34.6, 37.9)		18	42.4 (40.6, 44.3)	
Others	27	39.2 (37.0, 41.4)		10	42.4 (40.0, 44.8)	

^1^ Work on construction or demolition of buildings containing asbestos-containing materials such as insulation. ^2^ Work producing asbestos-containing products such as cements, slates, and fabric. ^3^ Maintenance and repair of asbestos-containing buildings or equipment. ^4^ Asbestos extraction, conveyance, and grinding work.

**Table 4:** Regression coefficients for mean latency periods by continuous exposure indicators after adjusting for covariates.

Variable	Malignant Mesothelioma		Lung Cancer	
	B (95% CI)	*p*-Value	B (95% CI)	*p*-Value
Occupational exposure				
Exposure duration (years)	−0.068 (−0.156, 0.019)	0.126	0.001 (−0.049, 0.052)	0.960
Age of first exposure (years)	−1.023 (−1.121, −0.925)	<0.001	−0.990 (−1.044, −0.936)	<0.001
Lifetime smoking (in pack years)	−0.020 (−0.059, 0.020)	0.325	−0.004 (−0.030, 0.023)	0.775
Environmental exposure				
Distance (km)	0.854 (−0.071, 1.779)	0.069	0.397 (−0.085, 0.878)	0.106
Exposure duration (years)	−0.018 (−0.135, 0.099)	0.755	0.000 (−0.045, 0.046)	0.987
Age of first exposure (years)	−0.985 (−1.123, −0.847)	<0.001	−0.960 (−1.014, −0.906)	<0.001
Lifetime smkoing (in pack years)	−0.017 (−0.066, 0.032)	0.484	−0.001 (−0.041, 0.039)	0.974

## Data Availability

The data presented in this study are available upon request from the corresponding author. The data are not publicly available because they contains sensitive patient information and location data.
